# Monitoring linear accelerators electron beam energy constancy with a 2D ionization chamber array and double‐wedge phantom

**DOI:** 10.1002/acm2.12751

**Published:** 2019-10-21

**Authors:** Song Gao, Mikhail A. Chetvertkov, William E. Simon, Amir Sadeghi, Peter A. Balter

**Affiliations:** ^1^ Department of Radiation Physics The University of Texas MD Anderson Cancer Center Houston TX USA; ^2^ Sun Nuclear Corporation Melbourne FL USA; ^3^ Radiation Oncology Banner MD Anderson Gilbert AZ USA; ^4^ Present address: Radiation Oncology Allegheny General Hospital Pittsburgh CA USA

**Keywords:** beam energy constancy, ionization chamber array, quality assurance

## Abstract

Validate that a two‐dimensional (2D) ionization chamber array (ICA) combined with a double‐wedge plate (DWP) can track changes in electron beam energy well within 2.0 mms as recommended by TG‐142 for monthly quality assurance (QA). Electron beam profiles of 4–22 MeV were measured for a 25 × 25 cm^2^ cone using an ICA with a DWP placed on top of it along one diagonal axis. The relationship between the full width half maximum (FWHM) field size created by DWP energy degradation across the field and the depth of 50% dose in water (R_50_) is calibrated for a given ICA/DWP combination in beams of know energies (R_50_ values). Once this relationship is established, the ICA/DWP system will report the R_50_FWHM directly. We calibrated the ICA/DWP on a linear accelerator with energies of 6, 9, 12, 16, 20, and 22 MeV. The R_50_FWHM values of these beams and eight other beams with different R_50_ values were measured and compared with the R_50_ measured in water, that is, R_50_Water. Resolving changes of R_50_ up to 0.2 cm with ICA/DWP was tested by adding solid‐water to shift the energy and was verified with R_50_Water measurements. To check the long‐term reproducibility of ICA/DWP we measured R_50_FWHM on a monthly basis for a period of 3 yr. We proposed a universal calibration procedure considering the off‐axis corrections and compared calibrations and measurements on three types of linacs (Varian TrueBeam, Varian C‐series, and Elekta) with different nominal energies and R_50_ values. For all 38 beams on same type of linac with R_50_values over a range of 2–8.8 cm, the R_50_FWHM reported by the ICA/DWP system agreed with that measured in water within 0.01 ± 0.03 cm (mean ± 1σ) and maximum discrepancy of 0.07 cm. Long‐term reproducibility results show the ICA/DWP system to be within 0.04 cm of their baseline over 3 yr. With the universal calibration the maximum discrepancy between R_50_FWHM and R_50_Water for different types of linac reduced from 0.25 to 0.06 cm. Comparison of R_50_FWHM values and R_50_Water values and long‐term reproducibility of R_50_FWHM values indicates that the ICA/DWP can be used for monitoring of electron beam energy constancy well within TG‐142 recommended tolerance.

## 1 INTRODUCTION

Periodic quality assurance (QA) of a clinical linear accelerator (linac) is important for dose accuracy during the treatment series of fractions. Deviations from baseline values acquired during commissioning^1^, ^2^ or from the treatment planning system (TPS) should not exceed the TG or MPPG tolerance levels. Both AAPM Task Group 142^2^ and AAPM Medical Physics Practice Guideline (MPPG8.a)^3^ recommend that electron beam energy constancy be evaluated monthly and annually. However, there are no recommendations on how the QA tests should be performed and what equipment should be used. The electron beam energy quality metric, “R_50_Water,” represents the depth at which the dose absorbed in water along the central beam axis, is reduced to 50% of its maximum value under full scatter conditions.^4^ Electron beam energy constancy is traditionally measured with solid water slabs during monthly QA. This procedure can be time consuming and becomes tedious on a linac with multiple electron energies as different thicknesses of solid water slabs are required to characterize each electron energy. In addition, a recent study has shown that the tolerances for electron beam energy checks using a two‐depth method are highly nonlinear due to the differences in gradient of the percent depth dose (PDD) falloff region.^5^

The concept of monitoring electron energy with a combination of a wedge and a linear detector has been extensively studied. The first published work^6^ was by Moyer in 1981 who used an aluminum wedge placed on top of a radiographic film to measure electron‐beam energy constancy for beam energies from 6 to 18 MeV and showed an overall uncertainty of ±0.4 MeV. Rosenow et al. used one‐dimensional (1D) ionization chamber array combined with a simple wedge‐shaped polystyrene phantom to measure beam energies. They found that the full width at half maximum (FWHM) of the modified electron profiles correlated linearly with R_50_ in the whole range of energies studied.^7^ Using the same 1D ionization chamber array and similar wedge‐shaped polystyrene phantom, Islam et al.^8^ were able to reproduce depth ionization curves in polystyrene phantom and the results agree quite well within water measurements for beam energy ≤10 MeV. Wells et al. used a home‐made double‐wedge acrylic phantom placed on top of a 1D diode array, and they found that the sensitivity of the combination of the diode array and double‐wedge technique is similar to water‐based depth‐dose measurements.^9^ Other similar work has been done more recently using linear arrays combined with wedges for electron beam energy determination.^10^, ^11^The extensive studies published over the years on monitoring electron energy with wedge shape attenuators did not result in changes in clinical practice.

In this work, we validated a commercial system for monitoring changes in electron beam energy based on the principle of a wedge‐shaped attenuator. The goal of this work was to ensure that a change in practice to use this device we will still meet the quality standards set out by the AAPM for electron beam energy stability (<2 mm uncertainty) while increasing our efficacy in performing QA. The system we are evaluating measures an electron beam energy constancy metric R_50_ using a two‐dimensional (2D) ionization chamber array (ICA) (IC PROFILER, Sun Nuclear, Melbourne, FL) with an aluminum double‐wedge plate (DWP) positioned on top of the ICA and the wedges along the positive diagonal axis. The double‐wedge electron profiles are invariant to phantom alignment in the wedge direction for a given ICA/DWP combination. The relationship of the distance between the 50% dose points under each wedge, FWHM, is related to the R_50_ values in beams of known energies. Once this relationship is established the ICA/DWP system will report a beam energy which we will be referred to as R_50_FWHM directly. We calibrated the R_50_ baseline with the ICA/DWP on a linear accelerator with energies of 6, 9, 12, 16, 20, and 22 MeV.We measured R_50_FWHM with the ICA/DWP for the calibration beams and also for beam energies lowered by using plastic sheets to shift beam energies. In addition R_50_FWHM data collected on a monthly basis on our clinical beams for a period of 3 yr during routine QA were used to evaluate the long‐term stability of both the device and out beam energies.

We noted that the manufacture's calibration procedure neglected the off‐axis ratio creating a calibration that was linac and beam dependent. We hypothesized that by removing the off‐axis ratio from the calibration, subsequent measurements could make the calibration universal. To study this we used an alternative calibration procedure for the ICA/DWP. Instead of normalizing the double‐wedge profile to the CAX, we normalized the double‐wedge profile obtained along the positive diagonal axis to the unwedged profile along negative diagonal axis. We compared calibrations and measurements on three types of linacs (Varian TrueBeam, Varian C‐series, and Elekta) with different nominal energies and R_50_ values.

The goal of this study was to evaluate the reproducibility of the electron beam energy metric R_50_FWHM measured with the ICA/DWP. We examined the uncertainties and stability of the R_50_FWHM values for known and unknown electron beam energies measured with the ICA/DWP and compared with measurements made with water scans. Measured R_50_FWHM compared to the measured R_50_Water showed good agreement for all electron beams in the study We demonstrated that the ICA/DWP can be used for monitoring of electron beam energy constancy with high accuracy (<1 mm error) and reproducibility (<1 mm variation).

## 2 MATERIALS AND METHODS

**Figure 1:**
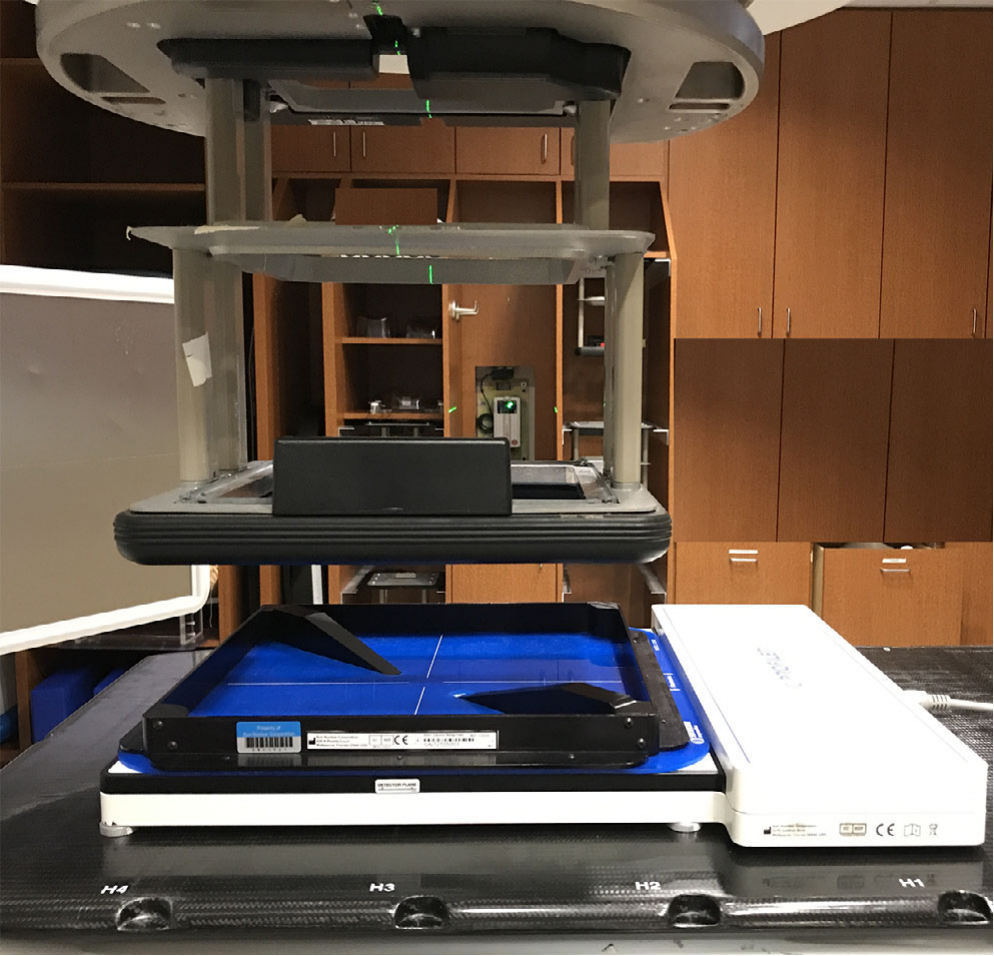
Ionization chamber profiler with double‐wedge plate. This view shows an extended SSD to better visualize the double‐wedge plate but the nominal SSD would be used for measurement.

The 2D ICA used in this work is IC Profiler which has 251 ion chambers at an effective depth of 0.9 cm. The detectors are arranged along the four axes, cross‐plane (x), in‐plane (y), positive diagonal (PD), and negative diagonal (ND). The principal axes (x and y) are 32 cm in length with 0.5 cm detector spacing, while the diagonal axes (PD and ND) are 45 cm in length with 0.7 cm detector spacing. Array calibrations which normalize the relative response of the off‐axis detectors to the central axis detector were performed initially on acceptance of the device and checked or repeated annually as per the manufactures’ recommendation.^12^, ^13^ The double‐wedge used in this work is a pair of aluminum wedges affixed at opposite ends of the positive diagonal axis mounted on an acrylic plate which is placed on top of the ICA based on alignment marks (Sun Nuclear Corp.). The aluminum wedges are 10.7 cm long and have a height of 3.1 cm (Fig. 1). With the DWP in place on the ICA, the distance between the narrow ends of the wedges is 7.5 cm, which leaves the central axis of the beam unattenuated on the ICA. Prior to use of the array we validated the accuracy of the array calibration to be within a 0.5% maximum point‐to‐point deviation using published methods.^14^

### 2.1 Calibration of electron beam energy metric R_50_

**Figure 2:**
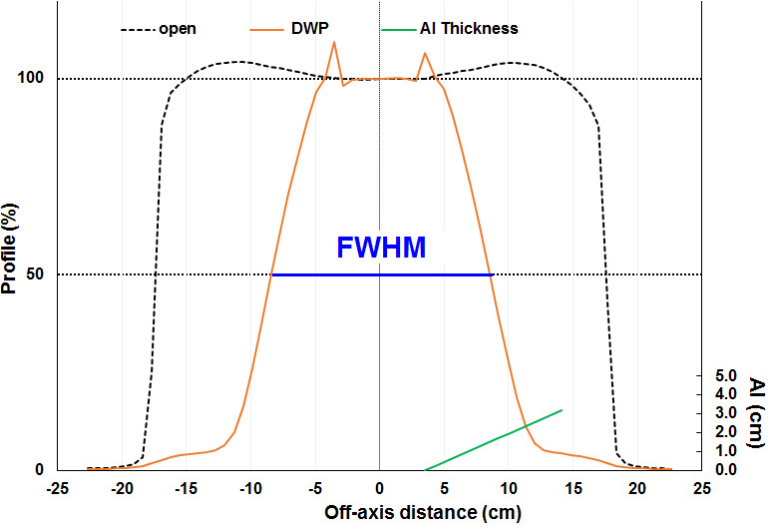
A comparison open field profile and the profile measured with ionization chamber array (ICA) and double‐wedge plate for 12 MeV electron beam. The full width half maximum of the profile at the 50% level was determined by IC Profiler software using linear least‐squares fit. The aluminum thickness of the double wedge in one side indicates the effect to profile.

In profiles acquired with a double‐wedge, the off axis distance (OAD) is directly related to the amount of aluminum that the beam has penetrated to reach the detectors. Thus the OAD that results in the signal being reduced to 50% of the value on the open central axis (OAD_50_) can then be directly related to beam energy. Rather than using the OAD_50_ directly the ICA uses the full width at half maximum (FWHM) of the diagonal profile as its energy metric (Fig. 2). This minimizes the uncertainties associated with device setup. The relationship between R_50_ in water and FWHM for each ICA/DWP combination is calibrated by the user in their electron beams by acquiring double wedge profiles and determining the FWHM in a number of beams with known R_50_. A linear fit is determined between FWHM and R_50_ which can then be used to measure R_50_ on beams from linear accelerators of the same design (make/model). We performed the calibration procedure on a linear accelerator (TrueBeam, Varian Medical Systems, Palo Alto, CA, USA) with nominal energies of 6, 9, 12, 16, 20, and 22 MeV. For each beam used in calibration we captured a profile in a 25 × 25 cm^2^ cone at 100 cm SSD with the DWP in place and associated this curve with the R_50_ for the same field. The data are used to determine a linear fit of R_50_ vs FWHM (*x*) [Eq. (1)].

(1) $$R_{50}=mx+b$$

The ICA software (IC PROFILER Software V3.4 and later) has the calculation of the R_50_ to FWHM built in so once the relationship is established an R_50_ value is reported directly to the user.

### 2.2 Measurement of R_50_ with ICA/DWP and verified with water scans

All measurements were taken with the ICA/DWP at SSD of 100 cm with a 25 × 25 cm^2^ electron cone. We measured R_50_FWHM values on the same linac with both the electron beams used for the calibration and with additional energies created by placing range shifters consisting of 0.075‐, 0.150‐ and 0.225 cm solid‐water in the beam path. To evaluate the accuracy of electron energy measured with the ICA/DWP, we compared R_50_ determined with the ICA/DWP to that measured in water from PDD scans for the nominal electron beams (6‐, 9‐, 12‐, 16‐, 18‐, 20‐, and 22‐MeV) as well as the unknown energy beams. In addition, we measured the R_50_FWHM on two other TrueBeam linacs with energies of 6, 9, 12, 16, and 20 MeV, and compared the ICA/DWP R_50_Water values with those determined from water scans.

### 2.3 Reproducibility of R_50_ measured with ICA/DWP

To test the short‐term reproducibility of the R_50_ measurement with ICA/DWP, we performed the measurement of each of the five electron beams on our Varian TrueBeam three times on the same day and analyzed the variation of the measurements. To verify the long‐term reproducibility of ICA/DWP for monitoring electron beam energy, we measured R_50_ on a monthly basis for a period of 3 yr.

### 2.4 Calibrations and measurements of R_50_ across types of linac

#### 2.4.1 Individual calibration and universal calibration

With the same calibration procedure and same measurement setup done on the TrueBeam (TB1) linac, we performed individual calibration on two other TrueBeam (TB2 and TB3), two Varain C‐series linacs (Clinac1 and Clinac2), and two Elekta (Elekta, Inc. Stockholm, Stockholm, Sweden) linacs (Versa and Infinity), with different nominal energies and R_50_ values (Table 1). The calibration results were exported from the ICA software and compared.

**Table 1:** The R_50_Water (cm) corresponds to the nominal beam energy for different types of the linac in this study.

Energy (MeV)	4	6	7	9	11	12	15	16	18	20	22
TrueBeam	×	2.39	×	3.53	×	4.94	×	6.60	×	8.27	8.78
Clinac 1	×	×	3.22	3.83	4.52	×	×	6.40	×	8.39	×
Clinac 2	2.07	2.65	×	3.84	×	5.08	×	6.49	×	×	×
Versa	×	2.58	×	3.70	×	4.93	5.98	×	×	×	×
Infinity	×	2.45	×	3.79	×	4.70	5.90	×	7.15	×	×

We also investigated a modified “universal” calibration and measurement procedure by taking the off‐axis ratios into consideration. This calibration procedure normalizes the positive diagonal double‐wedge profile to the unwedged negative diagonal profile. This was accomplished by exporting the ICA/DWP profile data to excel and manually calculating the FWHM values to establish the linear relationship between R_50_s and those FWHM values. This was done for each type of machine.

#### 2.4.2 Measurement of R_50_ with individual and universal calibration

We used both linac specific calibrations and the universal calibration protocol to determine R_50_ for a large number of beams on Varian TrueBeam and C‐series machines as well as on the Elekta Versa and Infinity machines. We measured 38 beams on the TrueBeam with energies over the range of 6–22 MeV including ones obtained with the range shifters used in Section 2.B. We measured ten beams on the C‐series machines with energies ranging from 4 to 20 MeV. We measured nine beams on the Elekta platforms with energies ranging from 6 to 18 MeV. For each type of machine we created an ICA/DWP calibration as per the vendor's instructions. We also created a universal calibration for each type of machine for cross comparisons of all measurements. For each machine we examined the accuracy of R_50_FWHM values determined using the calibrations obtained on that type of machine, calibration obtained on the other types of machines, and using the universal method.

### 2.5 Flatness and symmetry measurements with and without DWP

To assess the influence of the double‐wedge plate on the flatness and symmetry measurement in the principal axes we measured the profiles of nominal energy beams on the TrueBeam five times each with and without the double‐wedge plate on top of the ICA with the same setup on the same day. We obtained the flatness and symmetry values of each beam with and without DWP on ICA, then evaluated if the DWP affected the results by comparing the measured flatness and symmetry.

## 3 RESULTS

### 3.1 Correlation of the ICA/DWP profile with beam quality metric R_50_

The ICA/DWP calibration procedure was done using the 6, 9, 12, 16, 20, and 22 MeV electron beams on a Varian TrueBeam (TB1).The IC Profiler software determined a linear fit between the R_50_values in Table 1 and the measured FWHM of the acquired profiles. The linear approximation of R_50_Water and the FWHM (*x*) was: R_50_Water = 0.41154*x* – 1.90835 (R^2^ = 0.99975).

### 3.2 R_50_measured with ICA/DWP and compared within water scans

R_50_FWHM and R_50_Water measurements were made on all electron beams (6‐, 9‐, 12‐, 16‐, 18‐, 20‐, and 22 MeV) on the same linac used for the calibration, the measurements were repeated three times, each time with an independent setup. The maximum difference in the R_50_FWHM and R_50_Water values was 0.04 cm (Table 2). The reproducibility of the measurements was excellent (the standard deviation, σ ≤ 0.004 cm).

**Table 2:** R_50_Water (cm) vs R_50_FWHM (cm) for nominal energy beams, mean ± standard deviations (σ), and their difference: δR_50_ = R_50_FWHM – R_50_Water.

Energy	6e	9e	12e	16e	18e	20e	22e
R_50_FWHM (cm)	2.39 ± 0.004	3.53 ± 0.000	4.94 ± 0.004	6.59 ± 0.005	7.57 ± 0.004	8.26 ± 0.000	8.79 ± 0.000
R_50_Water(cm)	2.39 ± 0.002	3.53 ± 0.003	4.94 ± 0.003	6.60 ± 0.002	7.53 ± 0.003	8.27 ± 0.001	8.78 ± 0.002
δR_50_ (cm)	0.00	0.00	0.00	−0.01	0.04	−0.01	0.01

For beams not used for calibration and which were created by using different thickness of solid‐water as energy shifters, the maximum difference in the R_50_FWHM and R_50_Water values was less than 0.07cm (Table 3). The reproducibility of those measurements was similar to that in nominal energy beams.

**Table 3:** R_50_Water (cm) vs R_50_FWHM (cm) for energy shifted beams and their difference: δR_50_ = R_50_FWHM – R_50_Water.

Energy	6e	9e	12e	16e	18e	20e	22e
with 0.075 cm energy shifter, SD of δR_50_ (cm) = 0.024 cm							
R_50_FWHM (cm)	2.33	3.46	4.84	6.51	7.50	8.16	8.70
R_50_Water(cm)	2.32	3.45	4.86	6.52	7.45	8.19	8.71
δR_50_ (cm)	0.01	0.01	−0.02	−0.01	0.05	−0.03	−0.01
with 0.15 cm energy shifter, SD of δR_50_ (cm) = 0.032 cm							
R_50_FWHM (cm)	2.28	3.39	4.75	6.41	7.39	8.06	8.60
R_50_Water(cm)	2.25	3.38	4.79	6.45	7.37	8.12	8.63
δR_50_ (cm)	0.03	0.01	−0.04	−0.04	0.02	−0.06	−0.03
with 0.225 cm energy shifter, SD of δR_50_ (cm) = 0.031 cm							
R_50_FWHM (cm)	2.19	3.31	4.67	6.31	7.29	7.97	8.50
R_50_Water(cm)	2.19	3.31	4.72	6.37	7.30	8.04	8.56
δR_50_ (cm)	0.00	0.00	0.05	−0.06	−0.01	−0.07	−0.06

The R_50_ values of the five nominal energy beams on two other linacs (TB2, TB3) of the same model used for the calibration had identical results with the beams on the linac used for calibration. TheR_50_FWHM and R_50_Water ICA/DWP agreed within 0.04 cm (Table 4).

**Table 4:** R_50_Water (cm) vs with R_50_FWHM (cm) for nominal energy beams in other two TrueBeam linacs (TB2, TB3) and their difference: R_50_FWHM – R_50_Water.

Energy	6e	9e	12e	16e	20e
TB2, SD of δR_50_ (cm) = 0.022 cm					
R_50_FWHM (cm)	2.38	3.60	4.98	6.65	8.26
R_50_Water (cm)	2.38	3.59	5.02	6.65	8.30
δR_50_ (cm)	0.00	0.01	−0.04	0.00	−0.04
TB3, SD of δR_50_ (cm) = 0.024 cm					
R_50_FWHM (cm)	2.36	3.56	4.97	6.60	8.24
R_50_Water (cm)	2.32	3.54	4.99	6.62	8.24
δR_50_ (cm)	0.04	0.02	−0.02	−0.02	0.00

For all 38 beams of known and unknown energies for the same type of linacs, R_50_values measured using ICA with double‐wedge plate and those measured in water showed good agreement. The R_50_FWHM values agreed with R_50_Water of 0.01 ± 0.03 cm (mean ± σ) and a maximum discrepancy of 0.07 cm.

### 3.3 Long‐term reproducibility of R_50_ measured with ICA/DWP

**Figure 3:**
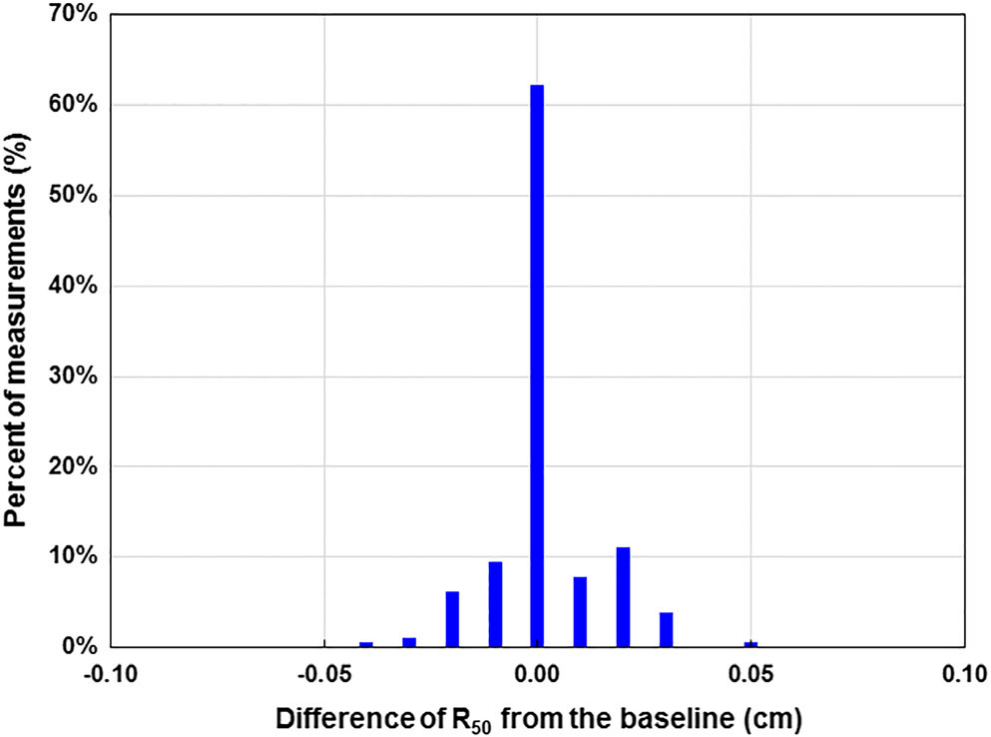
Histogram distributions of the differences of R_50_ measured with the ionization chamber array with double‐wedge and their baselines. These data represent 36 measurements for each of five electron beams. Measurements were performed on a monthly basis for a period of 3 yr.

Monthly measurements of R_50_FWHM on a single linear accelerator (TB1) for five electron beams over a 3‐yr period were used to determine long‐term stability. Histogram analysis of the R_50_ differences between the nominal R_50_ and R_50_FWHM indicated that 99.4% agreed within ±0.05 cm and 100% agreed within ±0.10 cm (Fig. 3). The standard deviations were <0.014 cm. (Table 5).

**Table 5:** The maximum difference (δR_50_) between ICA/DWP measured R_50_ and their baselines R_50_Water and the standard deviation (σ) of R_50_ measured with ICA for five nominal energy beams over a period of 3 yr.

Energy	6e	9e	12e	16e	20e
δR_50_ (cm)	0.040	0.020	0.010	0.020	0.020
σ (cm)	0.008	0.011	0.011	0.010	0.013

### 3.4 Calibrations and measurements of R_50_ across types of linac

#### 3.4.1 Individual calibration and universal calibration

**Figure 4:**
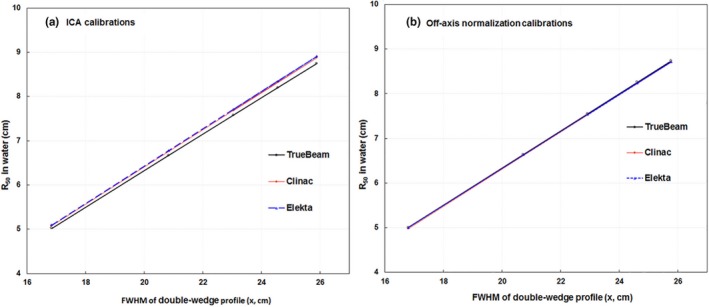
The R_50_ calibration based on the full width half maximum from the double‐wedge profiles of electron beams with known R_50_ values. (a) Individual calibrations from ionization chamber array software, (b) universal calibration by considering off‐axis corrections.

**Figure 5:**
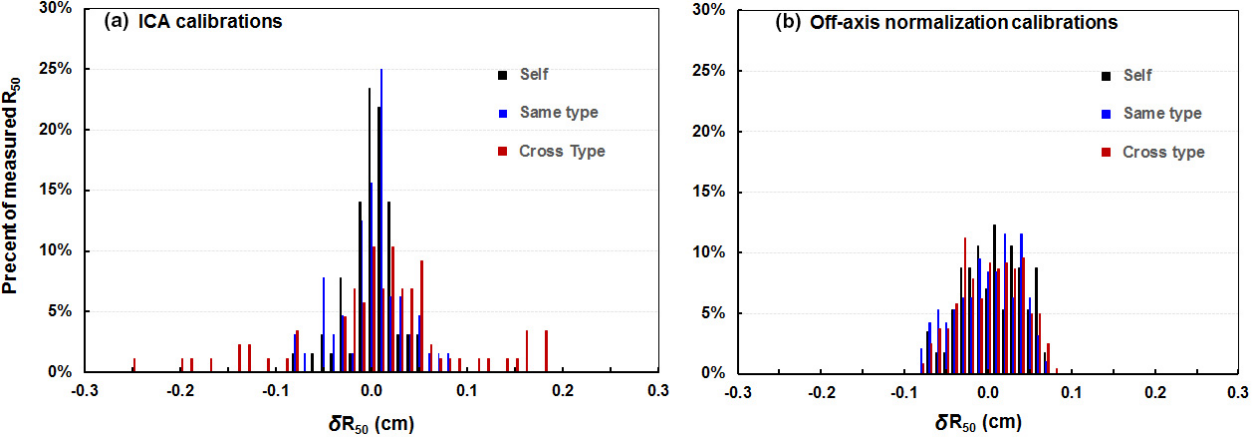
Histogram distributions of the differences in R_50_ full width half maximum with different calibrations and R_50_ water. “Self” indicates using the calibration determined on that particular linac, “Same type” is on a linac of the same maker and model but not the same machine, “Cross type” is using a calibration determined on a different maker and model of linacs. (a) Individual calibrations from ICA software, (b) universal calibration by considering off‐axis corrections.

The ICA/DWP calibration was performed on three Varian TrueBeams, two Varian C‐series with custom beam energies (Clinac1 and Clinac2) as well as on two Elekta machines, a Versa and an Infinity spanning a range of energies (Table 1). The slopes, intercepts, and Pearson coefficient for the calibrations for each linac was calculated by the SNC software (Table 6). Difference in the linear fits between the different linac types were dominated by the high‐energy beams [Fig. 4(a)]. The R_50_ values in water have good linear relationship with the FWHM. We only present data for energies ≥12 MeV to emphasize the differences of the ICA calibrations for different types of linac. For each beam on each linac we determined the discrepancy in R_50_FWHM vs R_50_Water when using the calibration done for that linac (Self), for another linac of the same type (Same Type), and for a linac of another type (Cross Type). We examined histograms of the differences [Fig. 5(a)] as well as quantifying the maximum difference for each linac across all beams. (Table 6). We found that “Self” calibration gave the best results with a maximum discrepancy of 0.07 cm, followed closely by “Same Type” calibrations with 0.08 cm with the worst results being “Cross Type” calibrations which had discrepancies of up to 0.25 cm.

**Table 6:** Individual calibrations from ICA software by linac type and their maximum difference: δR_50_max_ = R_50_FWHM – R_50_Water with different calibrations. “Self” indicates using the calibration determined on that particular linac, “Same Type” is on a linac of the same maker and model but not the same machine, “Cross Type” is using a calibration determined on a different maker and model of linacs.

Linac	Slope	Intercept	R^2^	δR_50_max_ (cm) per calibrations		
				Self	Same type	Cross type
TB1	0.4115	−1.9083	1.0000	−0.07	0.07	0.18
TB2	0.4181	−1.9876	0.9998	0.04	−0.06	0.15
TB3	0.4134	−1.9520	0.9996	0.00	0.05	0.15
Clinac1	0.4218	−2.0364	0.9993	−0.02	−0.08	−0.18
Clinac2	0.4194	−1.9833	0.9994	0.02	0.08	−0.11
Versa	0.4258	−2.0474	0.9999	0.00	−0.05	−0.25
Infinity	0.4214	−1.9991	1.0000	0.01	0.04	−0.20

The universal calibration that takes into account off‐axis corrections was also determined for each of these machines. The universal calibration resulted in the linear fits that were machine independent [Fig. 4(b)]. The discrepancies between R_50_FWHM and R_50_Water were slightly increased compared to the “Self” calibration, were equivalent to the “Same Type” calibration, and far superior to the “cross type” calibration [Fig. 5(b)]. With the universal calibration, the maximum discrepancy between R_50_FWHM and R_50_Water for “Self” and “Same Type” was modestly reduced to 0.06 cm while the maximum discrepancy for the “cross type” was dramatically reduced to also be 0.06 cm (Table 7) The improved agreement among slopes on the three linac types indicates the R_50_FWHM measurement method may be linac invariant if performed with universal calibration.

**Table 7:** Same as Table 6, but universal calibrations by linac types considering the off‐axis corrections.

Linac	Slope	Intercept	R^2^	δR_50_max_ (cm) per calibrations		
				Self	Same type	Cross type
TB1	0.4161	−1.9887	0.9997	−0.06	0.06	−0.06
TB2	0.4158	−1.9811	0.9998	0.05	−0.06	−0.03
TB3	0.4162	−2.0178	0.9999	0.05	−0.04	0.01
Clinac1	0.4160	−2.0256	0.9997	−0.05	−0.05	0.03
Clinac2	0.4155	−1.9998	0.9996	−0.05	−0.05	0.05
Versa	0.4163	−1.9889	0.9997	0.03	0.03	−0.02
Infinity	0.4139	−1.9493	0.9998	0.03	0.04	−0.03

### 3.5 Flatness and symmetry measurements with and without DWP

We measured flatness and symmetry of the principal axes (x and y axes) from the profiles acquired using ICA with and without the DWP. Five measurements were done for each case for each beam. The difference in measured flatness and symmetry with and without the DWP showed that the effects of the DWP were <0.10% on flatness and <0.15% on symmetry (Table 8). This is expected as the DWP does not block the detectors along principal axes.

**Table 8:** The differences of the flatness (δF) and symmetry (δS) present as mean ± standard deviation between five measurements without the double‐wedge plate (DWP) and those with DWP. Results are the average of in‐plane and cross‐plane.

Energy	6e	9e	12e	16e	18e	20e	22e
δF (%)	−0.10 ± 0.00	−0.04 ± 0.05	0.04 ± 0.03	0.00 ± 0.00	0.05 ± 0.00	0.00 ± 0.00	0.05 ± 0.00
δS (%)	−0.14 ± 0.05	0.08 ± 0.06	0.12 ± 0.04	0.07 ± 0.04	0.00 ± 0.00	0.11 ± 0.05	−0.01 ± 0.05

## 4 DISCUSSION

The traditional method of detecting changes in electron beam energy is by comparing the relative dose (signal) at a known depth (or thickness of plastic phantom) against a reference dose determined at the time of commissioning. This procedure is time consuming and becomes tedious for a multiple electron energy linac since different depths are required to characterize each electron energy. The flatness and symmetry measured from beam profiles are traditionally performed with 2D array (diode or ionization chamber) or film, which is another setup and measurement.

Measuring changes in R_50_ with an ionization chamber array combined with a double‐wedge gives equivalent result as in water scans without requiring the user to change depths between energies as well as providing the flatness and symmetry measurement simultaneously. Compared to other approaches using 1D/2D planner array,^5^, ^6^, ^7^, ^8^, ^9^, ^10^, ^11^ the method examined in this work has advantages: (a) the setup is easy and the baseline calibration is straightforward; (b) the Profiler software can directly report the value of R_50_; (c) the flatness and symmetry in principal axes and the beam energy R_50_ can be measured in one profile acquisition. We have also demonstrated that a modification of the calibration procedure to include the off‐axis corrections in the calibration would allow this ICA/DWP to directly determine R_50_ on different beams/machine types universally.

Furthermore, photon beam energy constancy metric, previous studies^15^, ^16^, ^17^ have demonstrated that an ICA can be used to measure changes in the energy, characterized by off‐axis ratio (e.g., in diagonal axes, diagonal normalize flatness, F_DN_) with a higher sensitivity than can be achieved with percentage depth‐dose measurements. Thus, we are able to replace both the photon and electron beam energy and profiles constancy measurements with a more efficient set of measurements using 2D IC array.

## 5 CONCLUSIONS

A convenient method for constancy checks of electron beam energies is described based on a commercially available ion‐chamber array and double‐wedge plate. In a single setup, this method is capable of measuring beam energy as well as the flatness and symmetry for electron beams. The results compared with in water measurement and the long‐term reproducibility indicated that the method of monitoring electron beam energy achieved an uncertainty level well below the clinical tolerance level of 2 mm. This combined with earlier published work on monitoring photon beam energy with similar device could be incorporated in routine QA procedures saving time while maintaining quality.

## CONFLICT OF INTEREST

The authors declare no conflict of interest.
